# Level and contributing factors of health data quality and information use in two districts in Northwest Ethiopia: social-ecological perspective

**DOI:** 10.1186/s12911-021-01741-1

**Published:** 2021-12-31

**Authors:** Binyam Tilahun, Lemma Derseh, Asmamaw Atinafu, Adane Mamuye, Tesfahun H. Mariam, Mesoud Mohammed, Teklehayimanot G. Hiwot, Berhanu Fikadie Endehabtu

**Affiliations:** 1 Department of Health Informatics, Institute of Public Health, College of Medicine and Health Sciences, University of Gondar, Gondar, Ethiopia; 2 Department of Epidemiology and Biostatistics, Institute of Public Health, College of Medicine and Health Sciences, University of Gondar, Gondar, Ethiopia; 3 Department of Health System and Policy, Institute of Public Health, College of Medicine and Health Sciences, University of Gondar, Gondar, Ethiopia; 4 Department of Computer Science, Faculty of Informatics, University of Gondar, Gondar, Ethiopia; 5 Policy, Planning, Monitoring and Evaluation Directorate, Ministry of Health, Addis Ababa, Ethiopia; 6 Amhara Regional Health Bureau, Bahir Dar, Ethiopia

**Keywords:** Incentive, Health data quality, Health data use, Ethiopia

## Abstract

**Background:**

The health management information system has been implemented at all levels of healthcare delivery to ensure quality data production and information use in Ethiopia. Including the capacity-building activities and provision of infrastructure, various efforts have been made to improve the production and use of quality health data though the result is still unsatisfactory. This study aimed to examine the quality of health data and use in Wogera and Tach-Armacheho districts and understand its barriers and facilitators.

**Methods:**

The study utilized a mixed-method; for the quantitative approach, institution-based cross-sectional study was conducted to determine the quality of health data and use by 95 departments in the two districts. The qualitative approach involved 16 in-depth interviewees from Wogera district. A descriptive Phenomenological design was used to explore factors influencing the quality and use of health data. The quantitative data were expressed descriptively with tables, graphs, and percent whereas the qualitative data were analyzed with content analysis guided by the social-ecological model framework.

**Result:**

The average levels of information use for Wogera and Tach-Armacheho districts were estimated at 29 and 35.9, respectively. The overall average level of accuracy of reports for six different health services in the HCs of Wogera and Tach Armacheho districts were 0.95 and 0.86, respectively.

The qualitatively identified factors that influence the production and use of quality health data include valuing data, getting staff training, being a patriotic staff, and getting supportive supervision, were identified from individual-level characteristics; similarly, coaching, supportive supervision, and peer-to-peer learning from relational/interpersonal level characteristics, and organizational culture, incentive, infrastructure establishing accountability, and staff turnover, were identified from organizational level characteristics.

**Conclusion:**

The quality of data and routine information utilization was low and were influenced by a number of actors presented in and around the health system including individual, interpersonal, and organizational characteristics. Incentive affects data quality and information use directly or indirectly after modifying factors at all levels of the social-ecological model. Therefore, interventions should gear towards addressing multiple social-ecological factors of the health system concomitantly or intervene on incentive which has a multifaceted effect on the outcome.

## Background

Quality healthcare data needs to be collected and stored at each level of the health system and are important to make informed decisions in the health system [1]. Utilization of poor quality data for routine health service delivery, planning and decision making could lead to a damaged health system that in turn would negatively affect both the individual and population health conditions [2].

In Ethiopia, health management information system has been implemented at all levels of healthcare delivery to ensure quality data production and information use [3]. Information use practice is the conscientious, explicit, and judicious use of current best evidence in making decisions about the care of communities and populations in the domain of health maintenance and improvement. The country has been implementing multiple strategies to enhance the performance of routine health information systems at different healthcare delivery levels as per the World Health Organization guideline [4] for a decade. Some of the strategies were related to steps made to advance the collection, aggregation, analyzing and reporting of health data [1]. Despite all those efforts, the generation of quality health data and its utilization are challenged by a number of factors that can be from inside or outside of the health system. As a result, the level of data quality and information use at each level of the health system is still unsatisfactory [5].

Therefore, relying on the usual trend can have little promise in addressing the identified gaps and may let the problem sustain for an extended period of time [6]. For any intervention to alleviate the identified problems of improving data quality and use, it is necessary to understand the current status of health facilities in regards to these parameters. It is also vital to explore the factors influencing data quality and information use which may be useful to overcome the barriers and capitalize on opportunities in meeting the aims set [5].

Factors influencing information utilization were completeness, consistency, relevance, and non-user friendliness of health information system (HMIS) formats and poor feedback from higher officials [3]. Other factors related to low healthcare data use are poor healthcare data management, poor management commitment, inadequate infrastructure, and high staff turnover [7–11].

A thorough examination of the factors that influence the generation and routine use of quality health data may depict the fact that they are from all actors presented in the health system. The factors may be associated with individual-level characteristics (personal characteristics of health workers, patients, managers, etc.), relationships/interpersonal level factors (related to events experienced by few and closest persons, e.g. peer-to-peer discussion), and organizational/community-level characteristics (e.g. organizational culture to data quality and information use and security in the community or civil unrest). Some of the factors such as using technology, incentive, and valuing data may be grouped under all three levels. This may imply the fact that factors influencing quality health data production and utilization can be assessed using the social-ecological model which was initially employed by the Centers for Disease Control and Prevention [12] so as to prevent violence.

**Fig. 1:**
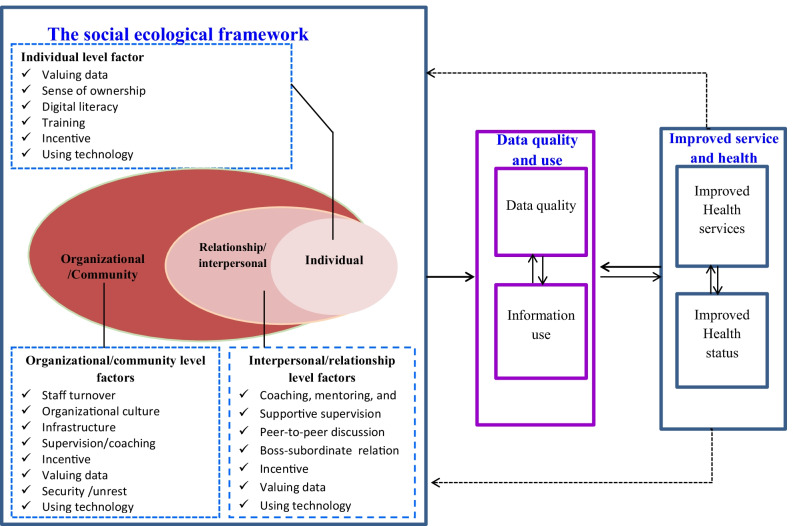
Conceptual framework to review the pathway of factors for the generation and use of quality health data. Source: A modified social ecological model of Dahlberg et al. [11] adapted for quality health data and use after literature review [3–5, 7–9, 11, 13–16]

In addition to quantifying the level of quality health data and its routine utilization, identifying the factors and understanding how they affect the quality of data produced and its utilization would be helpful to identify and design the strategies for future interventions. Therefore, the current study is designed to capture the characteristics of quality health data production and use in Wogera and Tach-Armacheho district and identify contributing factors using the social-ecological model (Fig. 1).

## Methods

### Study design and period

This study was based on mixed research approaches with triangulation design. Both the quantitative and qualitative components were given equal emphasis, and the two sets of results were converged during interpretation to draw valid conclusions about the research problem. For the quantitative approach, an institution-based cross-sectional study was conducted to determine the quality and use of health data generated in Wogera and Tach-Armacheho districts. On the other hand, the qualitative approach employed a phenomenological design to explore the factors that influence the quality and use of health data generated in the Wogera district from the lived experience of health workers. Both the quantitative and qualitative data were collected in 2020 concurrently.

### Study setting

The study was conducted in Wogera and Tach_Armacheho districts which are located in Northwest Ethiopia and are adjacent to each other. They were selected out of the 11 districts in Central Gondar Zone because, in addition to the government’s effort to improve data quality and use, other projects such as CBMP that work on the same task are distributed equally in these two districts [13]. Wogera district constituents 51 Kebeles and has a total population of about 278,942. There is 1 primary hospital, 8 health centers, and 44 health posts in the district which provides preventive, promotive, and curative services. Regarding the health workforce, there are 108 HEWs, 678 HWs, and 215 supportive staff. On the other hand, the Tach-Armacheho district has 24 kebeles with a population of 121,321. There is 1 primary hospital, 6 health centers, and 28 health posts. In addition, there are 53 HEWs, 202 HWs, and 141 supportive staff. The practice of producing and using quality health data in the study areas was low. Over-reporting and under-reporting of data elements were common in the primary health care unit of Wogera district [13].

### Participants, sample size, and sampling procedure

In the quantitative study, to assess the routine use of health data, the study population was the set of departments. In the data quality assessment, in examining the accuracy and completeness of data, records were elements of the study population, and for the quality assurance and availability of manuals assessment, health facilities are the unities of analyses. To get the experiences of data utilization among health workers, department heads were approached for the interview.

For the qualitative study, interviewees who had direct and indirect exposure to the generation and use of quality health data in Wogera district were the study participants. These participants were composed of both genders from different age groups and occupational, residential, and educational backgrounds. Specifically, it included heads of health centers, health information technicians, and department heads. In addition, key informants from the district health office were included.

For the quantitative component, 95 department heads (51 from Wogera and 44 from Tach-Armacheho districts) were included in the study. All departments that were functional during the study period were studied. For the qualitative component, 16 health workers participated in the interview which was determined after reaching information saturation. To be a part of the qualitative study, the potential interviewees need to be knowledgeable about the factors that influence the production and use of quality health data from different levels including individuals, relations/interpersonal (small groups of close persons), and organizational/community-level characteristics. The majority (10 out of 16) of the qualitative interviewees were also the respondents for the quantitative study.

### Data collection tools and procedures

After a structured questionnaire was drafted in both English and Amharic, the quantitative study tool was pretested with face-to-face interviews in Dabat district, and amendments were made accordingly. In the actual study, 3 data collectors in Wogera and 2 data collectors in Tach-Armacheho district health centers conducted the survey. The data collectors were given a two-day training including field practice with the questionnaire to help them be familiar with it. The data collectors chose suitable places to conduct the interviews where participants would be encouraged to respond freely, and records were also reviewed to get figures inquired. In addition, there was close and supportive supervision at the time of the survey.

To facilitate the qualitative data collection process, an open-ended, semi-structured in-depth interview guideline was prepared both in English and Amharic which mainly helped interviewees to tell how different factors influence the production and use of quality health data in their health facility. A pretest was conducted with the guideline in Dabat district, and a number of revisions were made on the wordings, content, and sequences of questions in the guideline. The in-depth interviews were conducted in the Amharic language by 2 interviewers who had ample experience in collecting similar data and had bachelor’s degrees. Moreover, tape recorders were used to record the interviews, and favorable places were provided to facilitate the confidentiality and quality of data and to address sensitive issues in the topics. The in-depth interviews took 30 min on average.

### Variables and measurement

The outcome variables for the study are the production of quality health data, and use for routine activities. Data quality characteristics including completeness, timeliness and consistency were assessed. The completeness of the data was assessed by measuring whether all the reportable data elements which are supposed to be reported actually did so. This was applied to health-centers reporting to districts’ health offices. To measure the discrepancy between reported and recounted data on the register, verification factor (VF) or data consistency was determined using the formula: VF = recounted from register divided by reported data. It was applied on data about six health service outcomes (ANC1, family planning, delivery, malaria, HIV + , and pneumonia) which were collected from the source document (register) independently and compared with the report. According to the national HMIS guideline, a VF value within 100% ± 10% is in the acceptable range. If the VF value > 110%, it indicates under-reporting, and if VF < 110%, over-reporting. Moreover, data quality assurance practice was determined by assessing Lots Quality Assurance sampling (LQAS) technique.

Information use was measured with five composite indicators including feedbacks, decision making using evidence, indicators identified, and health coverage calculated. Information use was reported in two ways: one was using the average value, and the other was by dichotomized at the average value to get “good” or “poor” outcomes.

From the qualitative component, various factors from individual, interpersonal/relationships, and organizational/community-level characteristics that were emerged during the in-depth interview and might affect the generation and use of quality health data were examined.

### Data management and analyses

Each copy of the quantitative questionnaire filled with data was checked for completeness before it was fed into the computer in Excel. Descriptive and summary statistics of the completeness, consistency, and accuracy of data entities were summarized.

The analysis of the qualitative data started in parallel with the data collection process as successive probing questions were raised based on participants’ responses. Data that were transcribed and translated to English were coded and organized thematically using the software Opencode4.03. To examine the factors that influence the generation and use of quality health data, the social-ecological model framework was employed as a guide in grouping findings to individual, interpersonal, and community/organizational levels. Thus, the method of analysis was content analysis, and each emerging theme was grouped in any of the three levels based on its relevance. It was also supported by direct (verbatim) quotations so that how and why the factors influence data quality and use could be understood better.

To enhance the credibility of the study, members of health facilities who had direct connections with the production and use of quality health data were recruited to participate. In addition, efforts were made to develop participants’ trust in the significance of the study so that open, complete, and truthful responses could be obtained using the appropriate data collection methods. The credibility of the study was further improved by triangulations such as mixed methods and prolonged engagement on the data which lasted for seven months. To maintain its transferability, detailed descriptions of the context of the study settings and participants were provided.

## Results

The present study involved all the 15 health facilities found in Wogera and Armacheho districts to examine the level of data quality and use. In addition, the entire 95 departments in the two districts were involved to assess their experience and perception in the generation or use of quality information. On the other hand, 16 health workers from Wogera district were involved to explore the factors that influence the generation and use of quality health data.

### Quantitative findings

#### Socio-demographic characteristics of participants

Of the 95 participants from the respective department heads involved in the study, 63 (66%) were males, 56 (59%) were 27 years or above in age, 64 (67%) were diploma holders in educational level, 20 (21%) were clinical nurse diploma by profession, 65 (68%) had 5 or less years of experience, and 49 (52%) were rural residents (Table 1).

**Table 1:** Socio-demographic characteristics of participants from Wogera and Tach-Armacheho districts, 2020

Variables	Wogera		Tach Armachiho	
	Number	Percent	Number	Percent
*Sex (N = 51, 44)*				
Male	29	57	35	80
Female	22	43	9	20
*Age in year (N = 51, 44)*				
< = 27	32	63	24	55
> 27	19	37	20	45
*Level of education (N = 51, 44)*				
Diploma	34	67	30	68
Bachelor	17	32	13	30
Master	0	0	1	2
*Profession (N = 51, 44)*				
BSC midwifery	4	7.8	2	4.5
BSC nurse	7	13.7	6	13.6
Health officers	3	5.9	3	6.8
Clinical nurse diploma	11	21.6	9	20.5
Health information technician	3	5.9	4	9.1
Laboratory diploma	5	9.8	4	9.1
Midwifery diploma	6	11.8	6	13.6
Pharmacy diploma	9	17.3	6	13.6
Others	3	5.9	4	9.1
*Experience in year (N = 51, 44)*				
< = 5	32	63	33	75
6–10	17	33	11	25
> = 11	2	4	0	
*Salary in ETB (N = 51, 44)*				
< = 5000	33	65	30	68
5001–10,000	18	35	14	32
*Work location (N = 95)*				
Urban	23	45	23	52
Rural	28	55	21	48

#### Facility and departments

Among 15 health facilities included in the quantitative study, 9 were from Wogera, and 6 were from Armacheho districts. Of the 9 health facilities found in Wogera district, 8 were health centers and 1 was a primary hospital. Similarly, 5 of the health facilities in the Tach-Armacheho district are health centers and the sixth one was a primary hospital. Of all departments involved in the study, maternal and child health (MCH) and pharmacy were each 9 (17.6%), out-patient department (OPD) 8 (15.6%), and emergency was OPD 5 (9.8%) (Table 2).

**Table 2:** Frequency of health facility characteristics of in Wogera and Armacheho districts, 2020

Variables	Wogera		Tach Armachiho	
	Number	%	Number	%
*Facility by type*				
Primary hospital	1	7	1	7
Health center	8	53	5	33
Total	9		6	
*Departments by type*				
Emergency OPD	5	9.8	4	9.1
Facility head office	3	6	6	13.6
HMIS unit	5	9.8	5	11.4
Laboratory	5	9.8	6	13.6
Maternal and Child health (MCH)	9	17.6	6	13.6
Out-patient department (OPD)	8	15.6	6	13.6
Pharmacy	9	17.6	6	13.6
Under five clinic	4	7.8	5	11.4
Others (ART, IPD, VCT)	3	6	0	0
Total	51		44	

#### Use of routine health information

The average levels of information use for Wogera and Tach-Armacheho districts were 29 and 35.9, respectively. In addition, the magnitudes of departments with information use above the average score were 39.2% and 45.5% for Wogera and Tach_Armacheho districts, respectively. The proportions of departments that gave feedbacks were 82.4% for Wogera and 68.2% for Tach-Armacheho districts. Decisions were made in 80.4% of Wogera and 72.7% of Tach-Armacheho departments; health coverage was calculated in 76.5% of Wogera and 70.5% Tach-Armacheho departments (Table 3).

**Table 3:** Routine health information use by health facilities in Wogera and Tach-Armacheho districts, 2020

Use of information	Wogera			Tach-Armacheho		
	Yes	No	Percent	Yes	No	Percent
Feedback given	42	9	82.4	30	14	68.2
Decision made using evidence	41	10	80.4	32	12	72.7
Health coverage calculated	39	12	76.5	31	13	70.5
Indicators identified	28	23	54.9	22	22	50.0
Target vs achieve calculated	31	20	60.8	26	18	59.1
Above the average information use	20	31	39.2	20	24	45.5

### Data quality

#### Level of accuracy

The overall average level of accuracy using recounted data from the registered and reported data in the HCs was 0.95 for Wogera and 0.86 for Tach Armacheho districts. Specifically, it was shown that for ANC1, the verification factor was 1.0 for month 1, 1.13 for month 2, and 1.01 for month 3 in Wogera district. In the same district, the three months average verification factor for ANC1 was 1.05. For ANC1, the accuracy level was 0.98, 1.1, and 0.83 for months 1, 2, and 3, respectively for Tach-Armacheho district (Table 4).

**Table 4:** Level of data accuracy in the health facilities of Wogera and Tach-armacheho districts, 2020

Health service/disease	Wogera				Tach Armachiho			
	Verification Factor_M1	Verification Factor_M2	Verification Factor_M3	Verification factor	Verification Factor_M1	Verification Factor_M2	Verification Factor_M3	Verification factor
ANC1	1	1.13	1.01	**1.05**	0.98	1.1	0.83	**0.97**
Family planning	1.01	0.84	0.98	**0.94**	0.93	0.83	0.76	**0.84**
Delivery	1.01	0.97	0.95	**0.98**	0.88	0.73	0.88	**0.83**
Malaria	0.75	0.88	0.89	**0.84**	1.1	0.98	0.86	**0.98**
HIV+	1.00	0.99	1.00	**1.00**	1.00	1.00	1.00	**1.00**
Pneumonia	1.03	0.96	0.63	**0.87**	0.65	0.58	0.43	**0.55**
Overall average verification factor				**0.95**				**0.86**

#### Completeness of data

Regarding the completeness of data, the discrepancy was 98 (3.6%) in Wogera district and 125 (5.6%) in Tach-Armacheho district.

#### Data quality assurance

Assessment of the quality assurance activities performed by health facilities showed that out of 15 health facilities, 12 performed self-assessment and 11 conducted LQAS. Out of 11 health facilities that conducted LQAS, 9 of them provided it for services reported in month-1, 9 for services report in month 2, and 10 conducted for services report in month 3 (Table 5).

**Table 5:** Quality assurance conducted by health centers in Wogera and Tach-Armacheho ditricts, 2020

Quality assurance	Frequency	
	Wogera (N = 9)	Tach-Armacheho (N = 6)
Self-assessment	7	5
Conducted LQAS	7	4
LQAS conducted for service report in Mont 1 (N = 11)	5	4
LQAS conducted for service report in Mont 2 (N = 11)	6	3
LQAS conducted for service report in Mont 3 (N = 11)	8	3
LQAS conducted for disease OPD report in Month 1	7	4
LQAS conducted for disease OPD report in Month 2	5	3
LQAS conducted for disease OPD report in Month 3	6	3

#### Timelines of reports

Of the 9 health facilities in Wogera district, the service report was submitted on time according to the national reporting guideline (20th to 26th of the month) for month 1 by 8 health facilities, for month 2 by 8, and for month 3 by 7 health facilities. In Tach-Armacheho district, the service report was submitted on time for month 1 by 6 health facilities, for month 2 by 5, and for month 3 by 4 health facilities out of a total of 6 health centers (Table 6).

**Table 6:** Timeliness of service and disease reports by Tach-Armachiho and Wogera districts, 2020

Indicators	Frequency	
	Wogera (N = 9)	Tach-Armachiho (N = 6)
Record submission date	7	5
Service report submitted on time for month 1	8	6
Service report submitted on time for month 2	8	5
Service report submitted on time for month 3	7	4
Disease report submitted on time by OPD for month 1	7	6
Disease report submitted on time by OPD for month 2	7	5
Disease report submitted on time by OPD for month 3	6	4

### Qualitative findings

Of the 16 participants involved in the in-depth interview, 15 were recruited from the health centers and 1 was from Wogera district health office. Among all interviewees, 15 of them were males, and their ages ranged from 24 to 38 years. Of the total participants involved in the study, 5 had the position of head of a health center, 6 HIS, and 4 were MCH, and 1 was head of Woreda health office (Table 7).

**Table 7:** Socio-demographic and work related characteristics of participants in Wogera district, 2020

Characteristics	Frequency
*Sex*	
Male	14
Female	2
*Age*	
20–25	4
26–30	11
30–40	1
*Occupation/position*	
HIT	6
MCH	4
Head of health center	5
Head of woreda HO	1
*Work experience (years)*	
2–3	4
4–5	5
6–7	5
8–9	2
*Work place*	
Health center	15
Woreda HO	1

Interviewees in the current study explained the methods of ensuring data quality by mentioning a set of criteria for determining the quality of a piece of information. Hence the criteria include accuracy, timeliness, completeness, and tangibility. However, in order to ensure the accuracy of the work done, they described that it is necessary to go down to the groundwork and check it. For example, cross-checking the data to ensure whether a certain mother has actually given birth in a health center is one approach mentioned by participants. And, cross-checking to state the quality of information and the accuracy of work done is another technique mentioned.

The current study mainly focused on assessing what factors influence the production and use of quality health data and how those factors affect both quality data generation and use in the context of Wogera district. The assessment revealed that there are a number of individual, relational, and organizational/community-level characteristics that affect the production and use of quality health data. In addition, effective use of data improves the health service delivery which usually results in improved health. In turn, understanding the significance of quality health data in improving the health of the community, those characteristics of individuals, relationships, and community/organizational would be modified in such a way that the production and use of quality health data will be improved.

The study revealed that the production and use of quality health data was not simply the influence of a few characteristics but the interwoven effect of factors from various domains. Thus, analysis of all characteristics that affect the quality and use of health data using the social-ecological model ended up with three themes. The three themes that emerged were related to individual, relational, and organizational level characteristics under which different factors were identified (Fig. 1).

### Individual level characteristics

#### Valuing data

In the present study, it has been shown that quality information is a primary transformation agenda that needs a high data quality revolution. It is also indicated that an organization is a real institution for the service it is established if all the work done is backed by data. Therefore, it is unlikely to deem that actual work is done if the respective data is not available. A respondent forwarded his opinion as, “*I can say work is done in our institution if properly reported to the relevant body, cross-checked and stored*” (Interview # 4, man); he also added saying “In our institution, it would be worthless if we claim we have indicators yet without data backing it.”

The significance of quality health data is highly acknowledged by participants. It is also understood that we have a history of poor data management though there are currently improvements or it is getting the attention of most actors. Supporting this idea a participant said, “The case team here understands that information is an important asset and a wealth to the country and for the community” (Interview # 1, man). He also added saying “*information is the evidence that reflects what we did*”. Considering that quality data helps to improve the health of persons and saves lives, a participant said that, “*As to me, data/information is a life*” (Interview # 3, man).

As evidence of valuing data, there are case team leaders in each health facility to monitor the quality of the health data at each unit during performance evaluation and they also check the data verification before sending it to the next level. An indication of good attitudes towards data is, quality service delivered to clients/patients and the data elements filled with the attributes of individuals who received the services need to be equal during crosschecking so that this can be an indication for a good attitude towards data. Attempts to recording, tallying, documenting all the data elements from patients and proper handling and reporting them are pieces of evidence for valuing data. In order to ensure those things, the person responsible has to always be in a state of readiness and ought to properly document any given information. A health worker who performs these activities regularly may develop a sense of ownership in the data recording and reporting process which is helpful to improve its quality. Patients or service seekers should also be cooperative for the generation of quality health data. “If a mother seeking health service gives a false age data, it does not only affect the treatment effect but also the data generated as well” (Interview # 1, man).

The availability of a performance management team (PMT) and striving for its responsibility are considered as evidence for valuing data. This can be confirmed from the regular meetings to evaluate the activities they performed in every month by PMT which helped some of the health workers to be role models in generating quality data. A respondent confirmed that “Even though health workers fall short in managing the data, we make sure it’s corrected in the next month. …Since there is a PMT and there is accountability now, I believe they are performing well” (Interview # 5, man). Evaluation of each other among health workers and provision of feedbacks regarding the health data are also mentioned as evidence of valuing data.

On the contrary, they also disclosed the prevailing problem of valuing and giving less attention to data, and it is due to the limitation health workers have in generating, handling, analyzing, and using health data. The poor attitude health workers have towards quality health data may be a factor that negatively affects the production and use of quality data and can be reflected in many ways such as carelessness, negligence, and being hurry to complete or register data properly which all have some link with valuing data.

The poor information culture that the health workforces have is also related to failing to value data. The value given for data is reportedly little and it is not really because of the lack of training but rather it is a failure to implement the training delivered properly. There was a lack of understanding on part of the person who has been given the responsibility. In this regard, a respondent said, “We are in an information age. We even have support from the University of Gondar. Generally it’s our lack of understanding and willingness that has become a barrier” (Interview # 4, man).

#### Getting training

Believing that training can facilitate the generation of quality data, capacitating staffs specifically on a basic computer or data handling and management was repeatedly proposed by respondents. In connection, lack of utilizing software can be an obstacle to the production and use of quality health data. Data sharing procedures such as sending and receiving information is easy using software, and lack of knowledge on software affects negatively the practice of health data. A respondent confirmed this saying, “… If training and basic infrastructures are put together, I believe we can achieve quality data use…” (Interview # 5, man).

However, study participants disclosed that the output of training including the development of information culture does not be seen soon. In this regard, a respondent said, “Quality data production and use may not be improved immediately yet we will see that in the process*”* (Interview # 5, man). On the other hand, another respondent suggested that waiting on site or offsite training to solve problems related to data production and handling is not good; instead, he recommended saying, “solving any issue by asking others who have a good performance during the morning session discussion which is a common practice in our office” (Interview # 7, man). The study also showed that training cannot necessarily maintain data quality for certain unless it has been implemented soon after its delivery.

#### Getting supportive supervision

The study identified that close and supportive supervision is vital for the generation and use of quality health data. In an environment where staff turnover is high, supportive supervision is mandatory after delivering relevant training. A 29-year participant argued as follows: “Saying do this and don’t do that couldn’t bring a change in the health system; rather coaching health care providers going where they are and observing what they work and showing on the spot is mandatory to bring quality in health data” (Interview # 15, man).

#### Being patriotic staff

The current study revealed that any person is said to be patriotic if he loves his country and if he loves what he does and serves his country with his profession. For health professions related to information, data must be generated, stored, and analyzed properly. It is worthless to work without properly storing information. In this context, a respondent said, “Someone who loves his country will do things carefully. If a person is patriotic, he needs to generate and store data carefully for future generation.” (Interview # 4, man). Another respondent substantiated the ideas saying, “a person who loves his country feels as if he killed his citizen or community whenever he produces false data” (Interview # 1, man).

An interviewee described how a patriotic person considers the establishment of a good health information system saying, *“As a patriot, you know that the information you have at hand is an invaluable resource for your country.* Even another interviewee emphasized that, “Just as you hold your mother in the highest regard, you ought to do the same for data because it is crucial for your country… By doing this, you greatly benefit your country” (Interview # 6, man). Another respondent explained what the modeled professionals in relation to data quality as, “By the way, there are people who are naturally inclined to doing good and who are attentive to information in general. Therefore they have taken the initiative to do what they do without expecting a reward” (Interview # 4, man).

### Relational/interpersonal level characteristics

#### Coaching and supportive supervision

Activities including teaching, facilitating, advising, advocating, and mentoring which are major tools of coaching are frequently mentioned as remedies to develop skill and motivation among the health workforce in generating quality health data and using it for planning and decision making. Supportive supervision is important especially after training to understand better and develop skills of quality data generation. A respondent explained his feeling saying, “Supervision and coaching should be followed with constant feedbacks ensuring its continuity.”

#### Peer-to-peer learning and mentoring

Peer-to-peer learning was raised as one of the preferred options to enhance the skill of producing quality data and its utilization; the justification for the preference is it easily opens peer discussion to reflect and evaluate one’s own practice on data and learn with a trusted professional in data management. It was argued that peer-to-peer discussions allow health workers to have others’ point-of-view on the matter which can help them develop their confidence in their practice. In addition to the ease to get peers both formally and informally, it has a strong social component which is one of its main strengths. A respondent explained his feeling saying, “For me, it is easy to discuss with peers and learn more; it is also easy to get them both in office or outside.” It has been mentioned that in addition to sharing knowledge, effective mentoring builds strong relationships within your organization.

#### Subordinate-supervisor relation

Working relationships between subordinates and managers both down and up the health facilities and offices make a vital difference in the effectiveness of data producers. The relationship between a health worker and his or her supervisor will have an effect on their level of motivation which in turn influences their performance. When data clerks feel connected to their supervisors, a conducive work environment would be created and thus enjoy their job more; they would also be loyal to their organization which would also reduce employee turnover which otherwise needs training new employees. “Good boss-subordinate relationship helps to develop a better working environment that encourages them to do their job in love; it also retains experienced staff longer which sustains the quality of data recordings.”

#### Staff turnover

The high turnover of health professionals was also mentioned as a factor influencing the production of quality data and use. In such a situation, unless constant training is given, the quality of data fails. A respondent stressed this saying, “When those who are trained staff goes and the untrained come, the quality of health data will be affected negatively” (Interview # 5, man)*.* They also proposed that as long as a new professional comes, the available senior staff should be responsible to train and coach the health professional.

#### Establishing accountability

In addition to other measures to improve data quality and use, it is also suggested to establish accountability across staff, departments, and organizations, and possibly in the community. A respondent explained the need for accountability saying, “It’s not just training that can solve this problem. In order for us to achieve high data quality, there needs to be accountability. Principles of accountability must be dispensed and people in that area must be critically held accountable” (Interview # 2, man). A respondent confirmed the reason why some health professionals did not work better and were not accountable for their poor performance saying, “Now the chain of accountability has been greatly weakened. Nowadays the person who does not take full responsibility for his work is not held accountable. Even more, it’s now considered a right to evade work; on the other hand, the one who does his work properly is considered a fool! We have not paid enough attention to developing a culture of accountability” (Interview # 4, man).

### Organizational characteristics

#### Infrastructure

It has been discussed that when information is handled in a traditional way, it is not readily available when needed. On the other hand, if the data management system is frequently upgraded and integrated with the latest technology, it would be easy to access data when inquired. By using the latest technology, it is easy to record and access any given information. Research can also be done based on the data at any given time and possible to store in soft than in hard copy and view them safely.

A respondent confirmed the significance of infrastructure saying, “if the health facility is not equipped with infrastructure like generator, we cannot use modernized methods or computerized works which affect the data quality” (Interview # 1, man). Another participant added saying, “If we have electric power, then information can reach us easily and quickly … If we have a network, we can communicate by e-mail; it reduces wastage of effort. Infrastructures can help us to shift from manual-based to computer-based works” (Interview #3, man). The establishment of internet connection is also mentioned as a factor to upgrade the knowledge of staff via Google and Facebook and so forth. However, trained personnel are needed to effectively use and capitalize on the infrastructures. On the other hand, another respondent claimed that infrastructure cannot be a concern to establish. In this context, a respondent said, “I don't think infrastructure is the challenge. … If we decide to set up infrastructure we have plenty of time to do it.”

Support from stakeholders is also mentioned as one reason for the improvement in data quality in health facilities. A respondent expressed how an institution works on data quality saying, “In the current context as the institution is supported by the University of Gondar, we are giving considerable attention for high data quality. I don’t reckon this institution has gaps in this area. We are actually working in keeping up with the current trend” (Interview #5, man). Frequent communications and meetings among HIT, PMT, woreda health office staff, and stallholders such as UoG were identified as major reasons for improvements in data quality.

#### Organizational culture

Organizational culture is mentioned as a reason for the generation of quality health data. Different structure team-ups in one-in-five, one-in-thirty, and other political structures are contributors to improved data. In this context, a respondent justified that, “One-in-five structures are very helpful to maintain data quality by evaluating their performance during their weekly meeting. One-in-thirty structures also evaluate their performance every two weeks and that helps to maintain data quality.”

#### Incentive

The study identified that in a health workforce, recognition can be relevant to establish a competitive environment among the health workforce. Rather than letting professionals become indifferent due to lack of recognition, at least it’s better to offer compensation based on the result they achieved. If possible, it is better to compensate with money or else give a certificate of recognition. Otherwise, health professionals might be discouraged. In this regard, a respondent said, “If we treat all together both hard worker and careless, it may demotivate hard workers and affect data quality” (Interview # 15, man). An interviewee explained the importance of incentives to boost the morale of health workers, saying, “If there is no any incentive, even a person can lose the data itself anywhere” (Interview # 2, man). Participants believed that incentives can motivate not only individuals but also higher levels such as health organizations. As individuals are incentivized, staff turnover can be minimized, may develop a sense of ownership for data quality, can value data, and develop a better organizational culture which all could result in better data quality and utilization.

However, the study identified that incentivizing staff was not a well-cultivated culture that might need more work to emphasize. In this context, a respondent explained his feeling saying, “There is no any experience of motivating professionals in our facility, though we have the plan to do it…” (Interview # 14, man). Of course, it has been argued that health professionals should work on health data effectively regardless of the provision of incentives. The same person expressed his feeling saying, “Everyone should be responsible for his/her duty regardless of the presence of incentive.” In this context, though incentive was not generally given emphasis to motivate staffs, some staff were still outshining in their duty regardless of it.

### Governance

Respondents argued that the improvements in the health services and health of persons have a great effect on the quality of health data to be produced and its utilization. If the health of the population is significantly improved, people would be motivated by the change and start listening and obeying what the healthcare providers are recommending. Supporting this idea, a respondent forwarded his feeling saying, “…If people see their health improved as a result of efforts by health workforce, then they would be motivated by the change and start cooperating with them including in data production…” (Interview # 2, man). However, the effect of the improvement of health services and health on the quality and use of health data mainly may not be a direct one. Instead, improved health services and health may modify the constituents of the social-ecological model which would intern affect the quality of health data and use. In this context, whenever people see improvements in health, they may start valuing data, be cooperative to give genuine information to healthcare providers and work seriously on data management and use, etc. which would result in quality data production and use.

Therefore, all direct contributors of improved health services and the health of persons are important for improved data quality and use. For instance, there may be feedback of relationships between the use of quality health data and the health of persons that can be worked on to improve each of them. The study identified that a quality health information system is beneficial not only for writing reports but also for the health of the community as a whole. For instance, one might report a case that does not exist which may create a problem to the health of the community. Respondents explained the link between quality health data and health-supporting with an example saying “For instance, for mothers on ANC, the health and growth of their children as well as their own health can be monitored for a better outcome if and only if we have quality data, and this will result in better health outcomes (Interview # 3, man).

Other contributing factors of health services and the health of persons such as regular supervision and monitoring of the routine work could improve service providers’ performance and services so that the linkage between health centers and other health facilities will be improved*. The absence* of strong linkage among health facilities could lead to inconsistency or fallacy between data report and data register. An interviewee reported that “Good data will bring a good service quality and promote patient satisfaction as well” (Interview # 8, man). It has been reported that based on the registers and tallies filled with clients' or patients’ information, one can identify weakness or poor performance so that we can improve it for the next service delivery processes.

## Discussion

The aim of this study was to determine the magnitude of and explore the factors influencing the quality and use of health data generated by health facilities. The average levels of information use for Wogera and Tach-Armachiho districts were found to be 29.0 and 35.9, respectively. The overall average level of accuracy of reports for six different health services in the health centers of Wogera and Tach Armacheho districts were 0.95 and 0.86, respectively. Various factors influencing the quality of data and use that range from individual to organizational level characteristics were identified. Among others, the factors included incentive, valuing data, sense of ownership, getting training, carelessness, coaching, peer-to-peer learning, supportive subordinate-supervisor relationship, staff turnover, and organizational culture.

In this study, the routine information utilization for the Tach-Armacheho district was lower than findings from studies conducted in East Gojam [14], Southern Ethiopia Hadiya zone [15], North Gondar zone [16], Eastern Ethiopia [9], and Arsi zone [17]; however, it is higher than findings from studies done in Jimma [18] and north Gondar zone [19] in Ethiopia. The possible justification for the variation in the reported estimates of data use might be related to the methodological differences used. In this regard, the East Gojjam, Hadiya, and North Gondar studies estimated the level of data use by health workers with percent; however, the current and Jimma studies estimated data use by health facilities by combining five types of data use. In addition, the variation could be the result of differences in data management knowledge, differences in the study period, and facility type/unit; zonal and district health offices were included in the case of Arsi and Jimma, and only the HIV/AIDS unit of health facility was studied in the case of Gondar, but in our case, all departments of health centers were studied. Whatsoever the reason for the variation of findings among studies, the utilization of data will be useful if and only if the quality of the data is good which otherwise will be damaging to the health and health system functioning.

The study revealed that the variation in the production and utilization of quality health data is due to differences in a number of factors clustering around the health facilities and workers. As a result, the discussion was framed in three groups including, individual, relationship/interpersonal, and organizational level factors.

### Individual level characteristics

Getting training improves the skill of the staff and helps them to develop a good attitude towards data/information which may be helpful to sustain the production and use of data quality. A study done in Nigeria showed that after an intervention of training, there was a significant increase in the completeness of reporting, overall accuracy rate, and timeliness rate of reporting [20]. Before effective training is given, health workers may have difficulty in filling registers or forms, and data analysis and use. So the training might resolve the difficulties and improve their performance in quality data production and use [20]. Training may also result in modifying positively the attitude of health workers so that they value information, which is identified as one factor influencing quality data generation and use in the current study [21]. Valuing data may give courage to them to produce quality data and to use it, especially when they understand the significance of data [14, 21]. As health workers value data, they would develop responsibility and a sense of ownership in their routine activities of data production, management, and utilization which would further improve data quality and use. The study also found out that meeting the goal of health facilities, i.e. improved health, can help health workers and the community to develop a better attitude towards data and valuing it, thereby facilitating the production of quality data and utilization [21]. This implies that there is a circular relationship among the social-ecological factors, quality health data production and use, and improved health.

On the contrary, the carelessness of both health workers and persons/patients who give data has an effect on data quality. A careless patient/client may give wrong data, or a careless health worker may record data wrongly as sufficient attention may not be given, and all these would affect the quality of data generated. Any public health decision or plan made based on wrong information will be misleading and usually ends up with poor health outcomes [2].

### Relationship/interpersonal level characteristics

Supportive supervision, coaching, or mentoring is helping to make things work, rather than checking to see what is wrong which is more common in controlling supervision. Supportive supervision is perceived as an intervention that strengthens the health system, enables health workers including health data management to offer quality data extraction and management, and improves performance [20, 22]. The traditional supervision approach has been understood to be unproductive, necessitating shifting to an easy approach that encourages joint problem-solving, mentorship, and communication between supervisors and supervisees [21, 23]. Supportive supervision typically includes performance observation and comparison of actual practices of data recording and management with standards; giving feedback on performance; provision of guidelines [24].

Direct interaction between peers who are working on health data recordings and management promotes active learning. Peers' discussion reinforces their own learning by instructing one another [21]. Peers feel more comfortable and open when interacting with each other, and they share a similar discourse about health and health management, allowing for a greater understanding of how to record, analyses, and store health data and use it.

### Organizational level factors

From the human resource perspective, staff turnover negatively affects organizational performance including data management and use because of loss of the knowledgeable, skillful and capable employees who have developed all these through experience and training [2]. Even if trained and skillful health personnel replaces the leaving staff, the health data management and use trend may not move, keeping its movements at least due to loss of memories. Unless training and supportive supervision are put in place after any turnover, the performance of the organization deteriorates severely. Of course, such problems can be minimized if a strong working system has already been established in the health organizations or health facilities [14, 21]. A well-established organizational culture of quality health data production and use is less prone to such turnovers and their consequences. Once producing quality health data and utilization for routine activities become the culture of health organizations, it would keep its momentum even at the time of difficulties [14].

Finally, incentive stands at all levels of the social-ecological model as it motivates individuals, small groups of persons (relationships) such as departments, and organizations or communities to get quality health data and use it for routine activities effectively. The incentives for respondents or patients may include opportunity costs [24] for health data recording and data management staff or individuals or organization; it can be a financial or in-kind incentive that motivates and take them to the next level [25]. In addition to the direct effect of incentive on the quality of health data and use, it can modify other factors mentioned at different levels of the social-ecological model which intern may affect health data quality and information use; in this regard, the incentive may help to develop a sense of ownership for health data quality, enhance valuing health data, facilitate peer-to-peer discussion, or minimize staff turnover, and improve organizational culture of producing and using quality health data.

### Limitation of the study

The quantitative study was based on small sample size, and as a result, we couldn’t measure the effect of different factors on quality data generation and use. However, efforts have been made to understand how different factors influence data quality and use qualitatively using the social-ecological model.

## Conclusion

The quality of data and routine information use was low, and were influenced by characteristics of all actors presented in and around the health system: individual (such as valuing data, incentive, getting training, developing a sense of ownership), interpersonal (coaching or mentoring, peer-to-peer discussion, boss-subordinate relationship), and organizational (staff turnover, organizational culture, and infrastructure). Therefore, interventions should gear towards addressing multiple social-ecological factors of the health system concomitantly or intervene on incentive which has a multifaceted effect on the quality of health data production and use.

## Data Availability

All relevant data are within the manuscript. The data upon which these findings were developed can also be available upon requesting the corresponding author.
